# Premenstrual dysphoric disorder and associated factors among female health science students in Wollo University, Ethiopia, 2017/18

**DOI:** 10.1186/s40748-019-0102-z

**Published:** 2019-05-21

**Authors:** Delelegn Tsegaye, Yemiamrew Getachew

**Affiliations:** 1 Department of Midwifery, College of Medicine and Health Sciences, Wollo University, P.O. Box 1145, Dessie, Ethiopia; 2 Department of Psychiatric Nursing, College of Medicine and Health Sciences, Wollo University, P.O. Box 1145, Dessie, Ethiopia

**Keywords:** Premenstrual, Dysphoric disorder, Dysmenorhea, University student

## Abstract

**Background:**

Premenstrual dysphoric disorder (PMDD) is also called late luteal phase dysphoric disorder. The syndrome involves mood symptoms, behavior symptoms and physical symptoms. This pattern of symptoms occurs at a specific time during the menstrual cycle, and the symptoms resolve for some period of time between menstrual cycles. It is one of the most common problems in female students of higher education institution that impaired academic performance and professional and interpersonal relationships. The main objective f this study is to assess Premenstrual dysphoric disorder and associated factors among Female health science students in Wollo University, east Amhara, Ethiopia, 2016/17.

**Methods:**

The study was conducted from January 1–15, 2017 involving 254 regular health science students were involved from college of medicine & health science, Wollo University. Institution based cross sectional study design was used. Systematic random sampling technique was utilized. Data were collected through interviewer administered standardized and pretested questionnaires. The collected data were presented in tables, graph & chart. Association between dependent and independent variable were tested using logistic regression model of SPSS version 20. Variables that have *P*-value less than 0.25 at bivarate analysis were entered to multivariate analysis model. Finally those variables which had P-value of < 0.05 were considered as having statistically significant association with the dependent variables.

**Result:**

The prevalence of premenstrual dysphoric disorder in this study was 66.9%. Degree of dysmenorhea was found to have statistically significant association with premenstrual dysphoric disorder. Students who had mild grade of dysmenorhea were less likely to have PMDD as compared with those students who had severe dysmenorhea (AOR = 0.13 at 95%CI (0.03–0.58). About 139 (61.8%) of female student reported that frequent class missing and low grade were occurred due to menstrual disorder.

**Conclusion:**

The Prevalence of premenstrual dysphoric disorder was highest as compared to other similar studies done in other countries. The factor associated with premenstrual dyphoric disorder was grade of dysmenorhoea. In order to tackle this problem, collaborative efforts should be taken.

## Introduction

Most of reproductive age women with regular menstrual cycles experience some symptoms in the luteal phase of their cycle [1]. In some women, these manifestations may be exaggerated and become a cause of misery, family disharmony, absenteeism, criminal acts like murder and suicide [1].

Premenstrual dysphoric disorder (PMDD) is a somatopsychic illness triggered by changing levels of sex steroid hormones that accompany an ovulatory menstrual cycle [2]. It occurs about 1 week before the beginning of menses and manifested by irritability, emotional liability, headache, anxiety, and depression. Somatic symptoms include edema, weight gain, breast pain, syncope, and paresthesias [2]. Approximately 5% of women have this disorder. Treatment of Premenstrual dysphoric disorder is symptomatic and constitutes analgesics for pain and sedatives for anxiety and insomnia [2].

Up to 90% of women of child bearing age are experienced by premenstrual symptoms but a smaller proportion of women meet criteria for premenstrual syndrome (PMS) and less than 10% are diagnosed as having premenstrual dysphoric disorder (PMDD). Premenstrual dysphoric disorder is a severe form of menstrual problem that manifests somatic, physical and emotional liability where as premenstrual symptom is minor problem that may occur in many females [3].

According to institution based cross-sectional study done in Bahir dar; the prevalence of dysmenorrhea and premenstrual syndrome were 85.1 and 72.8%, respectively. The contributing factors that were statistically significant and independently associated with premenstrual syndrome were educational status of mothers, living condition, having irregular menstruation and family history of premenstrual syndrome [4].

Therefore, this study was help to assess the effect of premenstrual dysphoric disorder in academic performance of female students in Wollo University which is found in Amhara region, Ethiopia. The result of this study will help to gain knowledge on PMDD problems & provide intervention. There was no study done on prevalence & effect of premenstrual dysphoric disorder in academic performance of female students in Wollo University which is found in Amhara region, Ethiopia. So it is important to deal on this issue because this problem has many negative impacts for female students who attend their education in higher institution.

The main objective of this study was to assess the Premenstrual dysphoric disorder and associated factors among regular Female health science students of Wollo university, east Amhara, Ethiopia. The specific objectives of this study were to assess prevalence of premenstrual dysphoric disorder & its associated factors among female health science students in college of medicine & health science in wollo University, 2017/18.

## Method and materials

### Study area and period

The study was conducted from February 1–15, 2017 in Wollo University. This University was established in 2007 and found in eastern Amhara region, Ethiopia. There were 582 undergraduate health science students in regular program during the study period.

### Study design

Institution based cross sectional study design was employed.

### Population

#### Source population

All undergraduate regular health science female students in Wollo University.

#### Study population

All undergraduate regular health science female students in Wollo University who were available in the campus during the study period.

### Inclusion and exclusion criteria

#### Inclusion criteria

Regular undergraduate health sciences female students who were registered in Wollo University in the first semester of 2016/2017 academic year.

### Sample size determination and sampling technique

#### Sample size determination

Sample size was determined by single population proportion formula and the required sample size was recruited from departments based on proportion to number of students in each department. 50% proportion of students with PMDD, 5% marginal error and standardized normal distribution at 95% CI were used to calculate the required sample size. Using those parameters the required sample size was calculated as;

$$ n={Z}_{\frac{a}{2}}^2\frac{P\left(1-P\right)}{d^2} $$

$$ \mathrm{ni}=1{.96}^2\times \frac{0.5\left(1-0.5\right)}{(0.05)^2}=384 $$

Where;

n- The minimum sample size required

P- Proportion of university students with PMDD

d- The margin of sampling error tolerated (5%)

Z _α/2_ the standard normal variable at (1-α) % confidence level and, α is mostly 5% i.e., with 95% confidence level.

The number of health science students was less than 10000, which were 582 students in regular programme so we have used correction formula.

$$ \mathrm{nf}=\mathrm{n}/1+\mathrm{n}/\mathrm{N} $$

$$ \mathrm{nf}=384/1+384/582 $$

$$ \mathrm{nf}=231 $$

For possible none response during the study the final sample size was increased by 10% to *n* = 231 + 10% which is 23 = 254

The final sample size was 254 students

#### Sampling techniques

Stratified random sampling technique followed by Systematic random sampling was utilized. Strata were established based on departments and sample frame was prepared using identification numbers of students taking from registrar office.

### Study variables

#### Dependent variables

Premenstrual Dysphoric Disorder.

#### Independent variables

- ✓ Socio-demographic variables
  - ▪ Age
  - ▪ Religion
  - ▪ Ethnicity
  - ▪ Relationship status
  - ▪ Distance from a place where family live
  - ▪ Resident where student currently reside
- ✓ Academic demand variables
  - ➢ Level of study year
  - ➢ Field of study
  - ➢ Interest to field of study
  - ➢ Students previous cumulative grade (CGPA)
- ✓ Family variables
  - History of severe discomfort during menstruation in the family
- ✓ Psycho-socioeconomic variables
  - ▪ Rank among the family to join university
  - ▪ Monthly pocket money
  - ▪ Life style(feeding carbohydrate foods)
- ✓ Menstrual cycle variables
  - Regularity of menstruation
  - Length of menstrual cycle

### Data collection procedures

#### Instrument

Data was collected using standardized measurement scale. Diagnostic criteria for PMDD was taken from diagnostic and statistical manual of mental illness 5th edition text revised (DSM-V TR). This instrument was developed by American psychiatric association and currently used in Ethiopia to diagnose clinical PMDD [2]. To assess other factors associated with PMDD among students we used pretested structured questionnaire developed after meticulous review of related literature.

#### Data collectors’ selection and training

Data was collected by three diploma nurses. Data collection tool were communicated with all data collectors during 1 day data collection training. One supervisor was assigned to follow data collection.

#### Data collection

The study participants were given a general introduction to the study as well as the opportunity to ask questions about the study and questionnaire were distributed. The principal investigator and the supervisor had checked the completed questionnaires for consistency and completeness on a daily basis.

### Data quality management

One day training for data collectors was given on how to collect data. The data collection methods, tools and how to handle ethical issues were discussed with the data collectors. Before the start of data collection for this study pre-test was conducted on 12 female students (5% of the sample size) who were not included in the main study to identify potential problems in the proposed study tool. Questionnaires filled during the pre-test did not included in the analysis as part of the main study. Amharic version of questionnaire was used. Regular supervision by the supervisor and principal investigator were made to ensure that all necessary data are properly collected. Each day during data collection, filled questioners were cheeked for completeness and consistency. The collected data were edited and processed timely and entered from a paper onto computer twice.

### Data processing, analysis, interpretation and presentation

Once all necessary data obtained, data were checked for completeness edited, cleaned, coded and entered in to and analyzed by SPSS version 20 for windows. Bivariate and multivariable regressions were used to identify the independent predictors of PMDD. This was done by entering each independent variable separately into bivariate analysis. Then, variables with *p*-value of less than 0.25 on bivariate analysis were entered into multivariate logistic regression. Then, variables which showed statistical significant association with p-value less than 0.05 in the multivariable logistic regression analysis were considered as predictors for PMDD.

### Operational definition

#### Students’ academic performance

Students’ academic achievement as measured using their CGPA in semester just preceding the study.

#### Premenstrual dysphoric disorder

Those women experience the following in the majority of menstrual cycles, at least five symptoms from diagnostic criteria of DSM-V TR must be present in the final week before the onset of menses, start to improve within a few days after the onset of menses, and become minimal or absent in the week post menses.

#### Regularity of menstruation

Those women whose menses flows every 28 days/4 weeks/.

#### Effect

Is the unwanted result related to bad feeling during premenstrual period.

#### Severe dysmenorhea

Subjective response of severe pain felt during menstruation.

#### Moderate dysmenorhea

Subjective response of moderate pain felt during menstruation.

#### Mild dysmenorhea

Subjective response of mild pain felt during menstruation.

### Ethical consideration

Ethical clearance was secured from Wollo University College of medicine and health science research review board and official letter will be communicated to University student affairs.

## Result

### Sociodemographic & reproductive characteristics of respondents

In this study 254 study participants were involved with a response rate of 100%. Of them, majority 216(85%) of the students were in the age group between 15 and 25 years. and the rest were aged above 26 years. About 109(42.9%) of respondents had height between 1.60 t0 1.69 m. Concerning their body mass index, most 199 (78.3%) of them had BMI with in the normal range (18–24). Regarding marital status of the respondents, 174(68.5%) were single, 36(14.2%) has boy friend and 43(16.9%) were married. With regard to their study year, most 109(42.9%) of the participants were 2nd year while 31(12.2%) 4th year. Regarding to the field of study most of them were nursing specialty student which were 97(38.2%). For 141(55.5%) of participants age at first menarche was between 13 and 15 years. In this study the average interval of menstrual cycle for 113 (44.5) study participants was regular (every 28 days) while 70(27.6%) reported average menstrual period interval of less than 28 days and the rest 71(28%) reported above 28 days.

Majority 133 (52.4%) of participants reported average length of menstrual period of 4–5 days of bleeding per one cycle while 10(3.9%) of respondents reported that they had menstrual bleeding formore than 8 days.

The amount of bleeding during one menstrual period as graded by the respondent were mild 29(11.4%), Moderate 191(75.2%) and heavy 30(11.8%) (Table 1).

**Table 1:** Sociodemographic and reproductive characteristics of female health science students in Wollo University, February 2017, Dessie, Ethiopia (*n* = 254)

Variables	Frequency(n)	Percentage (%)
Age in years		
15–25	216	85
> 26	38	15
Body mass index(BMI)		
Under weight (BMI < 18.50)	51	20.1
Normal BMI 18.50–24.99)	199	78.3
Over weight (BMI > 24.99)	4	1.6
Marital status		
Single	174	68.5
Has boy friend	36	14.2
Married	43	16.9
Widowed	1	0.4
study year		
1st year	85	33.5
2nd year	109	42.9
3rd year	29	11.4
> 4th year	31	12.2
Age at first Menarche (in years)		
< 13	12	4.7
13–15	141	55.5
16–18	93	36.6
> 18	8	3.1
Number of average days bleeding per one cycle in the last one year		
1–3	74	29.1
4–5	133	52.4
6–8	37	14.6
> 8	10	3.9
The amount of blood during one menstrual period		
Mild	29	11.4
Moderate	191	75.2
Heavy	30	11.8
Extremely heavy	4	1.6

As Table 2 showed most of the study participants 97(38.2%) were nursing students.

**Table 2:** Field of study of female health science students in Wollo University, February 2017, Dessie, Ethiopia (n = 254)

Field of study	Frequency	Percentage
Nursing(specialties)	97	38.2
Midwifery	46	18.1
Environmental health	16	6.3
Public health officer	30	11.8
Clinical pharmacy	31	12.2
Medical laboratory science	34	13.4

Nursing specialties (surgical nursing, operation theater nurse, Emergency & critical care nursing)

### Menstrual problems among students

Among study participants those who had dysmenorrhea (pain during menstruation) accounts 226 (89%) while the rest 28(11%) did not have pain during menstruation.

Of those who had dysmenorrhea 102 (40.2%) of them rate it as moderate, 77(30.3%) of them rate mild and 47(18.5%) reported severe (Table 3).

**Table 3:** List of symptoms & its grade on premenstrual dysphoric disorder among female health science students in Wollo University January 2017 Dessie, Ethiopia (n = 254)

Types of symptoms	Behavior or characteristics	Yes n(%)	No n(%)	Degree of symptom		
				Mild n(%)	Moderate n(%)	Severe n(%)
Physical symptom	Abdominal bloating	155(61)	99(39)	77(30.3)	62(24.4)	16(6.3)
	Breast tenderness	130(51.2)	124(48.8)	54(21.3)	63(24.8)	13(5.1)
	Generalized body pain	157(61.8)	97(38.2)	74(29.1)	61(24)	22(8.7)
	Headache	81(31.9)	173(68.1)	32(12.6)	36(14.2)	13(5.1)
	Back pain	142(55.9)	112(44.1)	43(16.9)	62(24.4)	37(14.6)
	Weight gain	45(17.7)	209(82.3)	32(12.6)	13(5.1)	45(17.7)
	Vomiting	24(9.4)	230(90.6)	12(4.7)	9(3.5)	3(1.2)
Emotional or psycho-behavioral symptoms	Eating more than usual	31(12.2)	223(87.8)	11(4.3)	16(6.3)	4(1.6)
	Anger	120(47.2)	134(52.8)	65(25.6)	29(11.4)	26(10.2)
	Craving for sweat foods, alcohol and appetite change	63(24.8)	191(75.2)	39(15.4)	18(7.1)	6(2.4)
	Forgetfulness	52(20.5)	202(79.5)	34(13.4)	12(4.7)	6(2.4)
	Loss of interest in doing things	143(56.3)	111(43.7)	55(21.7)	56(22)	32(12.6)
	Depressed mood	172(67.7)	82(32.3)	54(21.3)	76(29.9)	42(16.5)
	Sleep disturbances	123(48.4)	131(51.6)	47(18.5)	44(17.3)	32(12.6)
	Difficulty concentrating	105(41.3)	149(58.7)	43(16.9)	45(17.7)	17(6.7)

### Prevalence and perceived impacts of symptoms of premenstrual dysphoric disorder on students’ academic performance

**Fig. 1:**
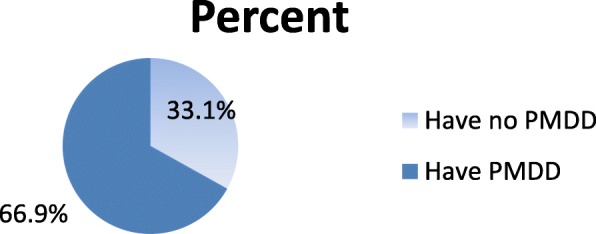
Prevalence of premenstrual dysphoric disorder among health science students 2017

In this study the prevalence of premenstrual disphoric disorder was 66.9% (Fig 1). Among those having PMDD 206 (81.1%) reported that the onset of symptoms was before they join university. While 23 (9.1%) started to experience symptoms after they join university. About 217(85.4%) of those having PMDD perceived that it had negative impact on their academic performance.

Majority of those who agree on negative impact of premenstrual symptoms on academic performance; majority 139(61.8%) reporting missed classes during the symptom. Six (2.7%) of those reported PMDD had history of withdrawal from school because of the symptom (Table 4).

**Table 4:** Perceived impacts of symptoms of PMS on students academic performance among female regular health science students in Wollo University, January 2017 Dessie, Ethiopia

Variables	Frequency(n)	Percentage (%)
When the above symptoms of PMS begin?		
Before joining university	206	81.1
In first year of university life	23	9.1
After first year of university life (2nd, 3rd,4th… year)	9	3.5
Do you perceive that the above symptoms have negative impact on your Academic performance?		
Yes	217	85.4
No	37	14.6
In what way symptoms cause impairment in your academic performance?		
Frequent class missing	139	61.8
Exam missing	16	7.1
Low grade scoring	57	25.3
Academic withdrawal	6	2.7
Others	7	3.1

Most of the students who had PMDD did not seek health care reporting fear of expressing symptoms related to menstrual cycle to health professionals. Others reported that they have considered symptoms as part of normal menstruation.

### Factors associated with premenstrual dysphoric disorder

Bivarate and multivariate logistic regression analysis were done to identify factors that are associated with premenstrual dysphoric disorder. On bivarate analysis the variables; residence, average length of one menstrual cycle, presence of dysmennorhea, grade of dysmennorhea, perception of PMDD as having negative impact on academic performance, perception of grade improvement if free from premenstrual symptoms and degree of bleeding during menstrual period had *p*-value of less than 0.25.

All variables whose *P*-value < 0.25 on bivarate analysis were entered to multivariate analysis model to identify variables which had association with PMDD. From the above variables only grade of dysmenorhea showed statistically significant associate with PMDD. Those students who had mild grade of dysmenorhea were less likely to have PMDD as compared with those students who had severe degree of dysmenorhea (AOR = 0.13 at 95%CI (0.03–0.58)) (Table 5).

**Table 5:** Factors associated with premenstrual dysphoric disorder among health science students of Wollo University January 2017 Dessie, Ethiopia (*n* = 254)

Variable	Premenstrual dysphoric disorder		Bivarate analysis		Multivarate analysis	
	No n(%)	Yes n(%)	COR	P. value	AOR (95% CI)	*P* value
Resident						
Live in the dormitory	71(31.6)	154(68.4)	1.76	0.15	1.55(0.61–3.96)	0.35
Outside dormitory	16(55.2)	13(44.8)	1		1	
Average length of one cycle						
< 28 days	37(26.2)	104(73.8)	2.0	0.01	1.65(0.88–3)	0.11
> 28 days	47(41.6)	66(58.4)	1		1	
Dysmenorrhea						
Yes	61(27)	165(73)	12.44	0.00	0	1.00
No	23(82.1)	5(17.9)	1		1	
Grade of dysmenorrhea						
Mild	59(33)	120(67)		0.001	0.13(0.03–0.58)	0.008^**^
Severe	2(4.3)	45(95.7)	1		1	
Have negative impact on academic performance						
Yes	61(28.1)	156(71.9)	4.2	0.00	2.36(0.95–5.89)	0.065
No	23(62.2)	14(37.8)	1		1	
Grade will be improved if free from premenstrual symptoms						
Yes	63(30.4)	144(69.6)	1.74	0.10	0.73(0.29–1.8)	0.51
No	19(43.2)	25(56.8)	1			
Amount of bleeding during menstrual cycle						
Mild & moderate	82(37.3)	138(62.7)	0.10	0.002	0.27(0.06–1.25)	0.096
Severe	2(5.9)	32(94.1)	1		1	

## Discussion

The prevalence of premenstrual dysphoric disorder among health science student in Wollo University was 66.9%. This finding is higher than study done in University student of Sistan and Baluchestan University (Iran) which was 36.3% [5, 7, 9, 10, 15, 18, 20]. The Possible explanation for this difference could be due to the sociodemographic characteristics of participant and the sample size difference. Study done in Mekele University, north Ethiopia, reported the prevalence of PMS was 37.0% [16] which was lower than ours. The difference could be due to variation in the tool to identify PMDD that Mekele’s study used diagnostic and stastical manual of mental illnesses 4th edition text revised (DSM-IV).

Study on prevalence and effect of Premenstrual syndrome on academic and social performances of students in Jimma University, Ethiopia indicated that almost all (99.6%) had at least one premenstrual (PM) symptom in many of the menstrual cycles in the past 12 months. The prevalence of premenstrual dysphoric disorder in this study was 27% which is lower than our study finding [17]. The possible reasons for this discrepancy could be sample size difference, data collection tool used, difference in study period.

The prevalence of PMDD in our study was 66.9% which is lower than study done in Isra University Hospital, Hyderabad, Sindh, Pakistan which was 88.7%, [6] study conducted in the college of Nursing, Hawler Medical University in Iraq reported that higher prevalence of premenstrual symptoms 81.3% [12] and other study done in Islamabad Army Medical College Pakistan, National University of Sciences and Technology Pakistan showed prevalence of PMDD 92.4% [8, 11]. The reason for this difference may be due to difference in study period, sample size, study design and data collection instruments.

In our study severity of dysmennorhea had statistically significant association with PMDD indicating those students who had severe pain during menstruation were more likely to experience PMDD as compared to those students who had mild degree of dysmenorhea. (AOR = 0.13 at 95%CI (0.03–0.58). This finding is similar with study done in Turkey, Pakistan, and Egypt [8, 11, 14] which also reported presence of PMDD significantly related with severe dismenorhea.

Even though PMS was significantly associated with older age groups, average length of one cycle of menstruation and academic performance impairment, rural residence, lower age at menarche, regularity of menses and family history in other studies [13, 16, 19] they showed no statistically significant association in this study.

### Strength & limitation of the study

#### Strength of the study

- using probability sampling techniques it will represent health science students
- large sample size

#### Limitation of the study

- Being cross-sectional study design, it will not show cause and effect analysis

## Conclusion

In this study the prevalence of premenstrual dysphoric disorder was high as compared with other studies done in Ethiopia and some other countries. Among factors assessed in this study only severe dysmenorrhea had statistically significant association with PMDD. Majority of students suffering from PMDD did not seek health care due to fear of reporting symptoms related to menstruation to health professionals.

### Recommendation

#### To Wollo University college of medicine & health science

- This problem had significant negative effect on female student academic performance so awareness creation to improve health care seeking behavior among students has to be designed and implemented.

#### To health care providers who are working at Wollo University student clinic

- Training for health care team in the clinic on assessment and treatment of PMDD in collaboration with department of mental health nursing

#### To research community

- Further studies should be carried out to understand the problem involving students in other colleges and institutions.
